# Improvement of Distribution and Osteogenic Differentiation of Human Mesenchymal Stem Cells by Hyaluronic Acid and β-Tricalcium Phosphate-Coated Polymeric Scaffold *In Vitro*

**DOI:** 10.1089/biores.2015.0021

**Published:** 2015-09-01

**Authors:** Muwan Chen, Dang Q.S. Le, Jørgen Kjems, Cody Bünger, Helle Lysdahl

**Affiliations:** ^1^ Orthopaedic Research Laboratory, Aarhus University Hospital, Aarhus, Denmark.; ^2^ Interdisciplinary Nanoscience Center, Aarhus University, Aarhus, Denmark.

**Keywords:** bone tissue engineering, cell distribution, human mesenchymal stem cell, osteogenic differentiation, scaffold

## Abstract

Bone tissue engineering requires a well-designed scaffold that can be biodegradable, biocompatible, and support the stem cells to osteogenic differentiation. Porous polycaprolactone (PCL) scaffold prepared by fused deposition modeling is an attractive biomaterial that has been used in clinic. However, PCL scaffolds lack biological function and osteoinductivity. In this study, we functionalized the PCL scaffolds by embedding them with a matrix of hyaluronic acid/β-tricalcium phosphate (HA/TCP). Human mesenchymal stem cells (MSCs) were cultured on scaffolds with and without coating to investigate proliferation and osteogenic differentiation. The DNA amount was significantly higher in the HA/TCP-coated scaffold on day 21. At the gene expression level, HA/TCP coating significantly increased the expression of *ALP* and *COLI* on day 4. These data correlated with the ALP activity peaking on day 7 in the HA/TCP-coated scaffold. Scanning electron microscope and histological analysis revealed that the cell matrix and calcium deposition were distributed more uniformly in the coated scaffolds compared to scaffolds without coating. In conclusion, the HA/TCP coating improved cellular proliferation, osteogenic differentiation, and uniform distribution of the cellular matrix *in vitro*. The HA/TCP-PCL scaffold holds great promise to accommodate human bone marrow-derived MSCs for bone reconstruction purposes, which warrants future *in vivo* studies.

## Introduction

Bone tissue engineering (BTE) and reconstructive surgery have been intensively researched in the past 20 years. The key challenge for successful BTE is creating an ideal scaffold having the following properties: a porous structure, high interconnectivity, adequate mechanical properties, biodegradability, biocompatibility, osteoinduction, and osteoconduction. The scaffold should support cell attachment, migration, proliferation, and differentiation and eventually be replaced by the regenerated host tissue.^1–3^

No single biomaterial accomplishes all the required properties of the BTE scaffold. Composite scaffold designs can take each material's advantage to fulfill most of the requirements and significantly improve the physical, chemical, and biological properties.^4–6^

Biodegradable polyesters, such as polyglycolic acid, polylactic acid, and polycaprolactone (PCL), are the most commonly used synthetic polymer materials for BTE applications. They are attractive candidates for use as scaffolds because they can be fabricated for a wide range of biodegradable biomedical applications with a high-process ability, controlled degradation, adjustable mechanical properties, and with the possibility of a wide range of modifications.^7^ Their degradation products are tolerated by the human body and can be removed by physiological metabolic pathways.

Clinically, PCL scaffolds made by fused deposition modeling (FDM) have been studied for more than 10 years and gained FDA approval in 2006.^8^ However, these FDM-manufactured PCL scaffolds comprise a macroporous structure, lack biological functionality in the scaffold–cell interface, and are inherently hydrophobic. To overcome such disadvantages, improvements by incorporating natural polymer and bioceramic material into the scaffolds have been performed: PCL scaffolds have been coated with natural polymers such as collagen,^9,10^ chitosan,^11^ hyaluronic acid (HA),^12^ and silk fibroin^13^ to improve cell affinity and biocompatibility.

Bioceramics, such as hydroxyapatite and β-tricalcium phosphate (TCP), are calcium phosphate products, which have often been used for BTE.^14,15^ However, their brittle and fragile properties limit their uses as defect fillers in orthopedic surgery. Therefore, blending bioceramics with PCL polymer to reduce the ceramic materials' intrinsic brittleness and to promote osteoconductivity of PCL material has been performed.^4,6,16^

We have previously shown that functionalization of PCL scaffolds with HA and TCP facilitates migration and osteogenic differentiation of human dental pulp stem cells *in vitro*.^17^ The aim of the current study was to investigate the osteogenic potential of this scaffold seeded with human bone marrow-derived mesenchymal stem cells (MSCs). We hypothesized that HA/TCP coating would promote distribution, proliferation, as well as osteogenic differentiation of human MSCs (hMSCs) *in vitro*.

## Materials and Methods

### Scaffold fabrication

FDM scaffolds were made from PCL with a molecular weight of 50 kDa (Perstorp) at a processing temperature of 106°C with a BioScaffolder (SYS+ENG GmbH). The stainless steel extrusion needle (DL Technology) had a 200 μm opening, which produced extruded polymer strands with a width of ∼190 μm. A square of 36 × 36 × 2 mm mat was made first, and cylindrical scaffolds with a diameter of 4 mm were punched out using a biopsy punch (Acuderm). To increase surface hydrophilicity and, thus, improve cell attachment, the scaffolds were treated in a 5 M sodium hydroxide bath for 3 h, neutralized by washing with phosphate-buffered saline (PBS) and sterile water, and disinfected using 70% ethanol. The scaffolds were rinsed in sterile water multiple times and dried. These scaffolds are hereafter referred to as PCL scaffolds.

HA/TCP-PCL scaffolds were fabricated as described previously.^17^ Briefly, the PCL scaffolds were soaked in the HA/TCP suspension for 12 h. HA/TCP suspension was prepared by dispersing 10 wt% TCP (BABI-TCP-N100, particle average size 100 nm; Berkeley Advanced Biomaterials, Inc.) into an aqueous 4 mg/mL HA solution (MW = 780 kDa; Lifecore Biomedical). Finally, these scaffolds were placed in a freeze dryer (FreeZone Triad Freeze Dry Systems) at −20°C, 30 mTorr, for 4 days.

### Scaffold characterization

The morphologies of the scaffolds were observed using a scanning electron microscope (SEM; Nova NanoSEM 600; FEI Company). Samples were observed at an acceleration voltage of 5 kV in the environmental mode. Energy dispersive X-ray spectrometer (EDX) was used to examine the distribution of the HA/TCP coat in the scaffolds based from the Ca^2+^ element in the TCP.

### Cell passaging and scaffold culturing

Bone marrow-derived hMSCs (PT-2501, lot 1F3284, Lonza), 21-year-old female donor, 11.42 population doublings (passage 5–7), were seeded at a density of 4000 cells/cm^2^ in culture flasks in Dulbecco's Modified Eagle's Medium (DMEM) without Phenol Red (21063; Gibco, Invitrogen)^18^ supplemented with 10% fetal bovine serum (FBS) and cultivated in a humidified atmosphere of 37°C and 5% CO_2_. After 1 week, cells were trypsinized and resuspended to 4 × 10^6^ cells/mL in DMEM with 10% FBS.

Scaffolds (PCL and HA/TCP-PCL) were placed in 24-well plates (one scaffold/well, lot 83.1836.500, Sarstedt). hMSCs were applied dropwise, 10 μL, on top of the scaffolds, 40,000 cells/scaffold, and incubated for 2 h at 37°C in a humidified atmosphere of 5% CO_2_ before the Osteogenic Differentiation Medium consisting of DMEM supplemented with 10% FBS, 100 nM Dexamethasone (D2915; Sigma), 10 mM β-Glycerophosphate (G9891; Sigma), 50 μM L-Ascorbic Acid-2 Phosphate (A8960; Sigma), and 10^−8^ M 1α,25(OH)_2_D_3_ (D1530; Sigma) was added 1 mL/scaffold. The cells were cultured for up to 21 days at 37°C in a humidified atmosphere of 5% CO_2_. The medium was changed twice a week.

### Evaluation of cell distribution and morphology within the scaffolds

Toluidine blue staining and SEM were used to visualize the cell distribution and morphology after 21 days of culture.

For Toluidine blue staining, scaffolds from each group were fixed in 70% ethanol, embedded in Technovit^®^ 7100 (Ax-lab), and vertically cut in sections of 25 μm using a Sawing Microtome KDG 95 (Meprotech). Sections were stained with 0.1% Toluidine blue (Fluka) at pH 7; pictures were taken from the central part of the scaffold and in the Z-direction from top to bottom using a BX50 microscope with a Camedia C-5060 camera (Olympus).

For SEM, scaffolds from each group were rinsed in PBS and fixed in 2.5% glutaraldehyde containing a 0.1 M sodium cacodylate buffer (pH 7.4), dehydrated in a graded ethanol series, and air-dried. Pictures were taken from both sides of the scaffold (cell seeding side and the bottom side) as well as cross sections.

### DNA quantification

DNA quantification was estimated by the Quant-iT^™^ PicoGreen^®^ dsDNA assay (Life Technologies). Cellular–scaffold constructs from each group (PCL scaffold and HA/TCP-PCL scaffolds) were transferred to 2-mL Eppendorf Tubes with 200 μL DMEM w/o Phenol Red and sonicated at intervals of 1 sec on/5 sec off with amplitude on 50% (0.046 kJ) for a total of 1 min. Sixty microliters of this solution was taken out to be used for the alkaline phosphatase (ALP) activity test. Then, collagenase (Sigma C8176, 100 mg/mL) was added 4.8 μL/tube and incubated at 37°C for 3 h with shaking before 7.7 μL Proteinase K (Sigma P2308, 20 mg/mL) was added and incubated at 45°C for 20 h. Scaffold debris was spun down at 10.000× *g* for 5 min.

Samples were prepared according to the manufacturer's protocol and analyzed using a microplate reader (Victor3 1420 Multilabel Counter; PerkinElmer Life Sciences). Technical duplicates were used for each biological sample. The rest of the solution with the cell–scaffold construct was continued and used for calcium contents assay.

### Osteogenic differentiation of hMSCs within the scaffolds

#### Total RNA extraction and reverse transcription quantitative polymerase chain reaction

Total RNA was extracted after 2, 4, 7, 14, and 21 days in osteogenic cultures with the GenElute^™^ Mammalian Total RNA Miniprep Kit (RTN 350; Sigma-Aldrich) according to the procedure of the manufacturer. RNA concentration and purity were spectrophotometrically determined using an IMPLEN NanoPhotometer^™^ (VWR Bie & Berntsen) according to the manufacturer's instructions. RNA integrity was determined using an Agilent 2100 Bioanalyzer (Agilent Technologies) according to the procedure of the manufacturer. The RNA samples were treated with DNase I (AM2222; Ambion) and converted into complementary DNA (cDNA) using the High-Capacity cDNA Reverse Transcription Kit (4368813; Applied Biosystems).

Reverse transcription quantitative polymerase chain reaction (RT-qPCR) was performed on a 7500 Fast Real-Time PCR system (Applied Biosystems) using the TaqMan^®^ Fast Universal PCR Master Mix (4366073; Applied Biosystems) and TaqMan Gene Expression Assays (4331182; Applied Biosystems) with the following assays: runt-related transcription factor 2 (*RUNX2*) Hs00231692_m1, *ALP* Hs00758162_m1, collagen type I alpha 1 (*COLI*) Hs00164004_m1, and bone gamma-carboxyglutamate protein (osteocalcin [*OC*]) Hs01587813_g1. Standard enzyme and cycling conditions for the 7500 Fast System were used. Template cDNA corresponding to 8 ng of RNA was added to each PCR, and each biological sample was run in technical duplicates for each gene. Data analysis was performed using 7500 Fast System Sequence Detection Software version 1.3 (Applied Biosystems). Based on BestKeeper,^19^ values were normalized to ubiquitin C Hs00824723_m1, beta-2-microglobulin Hs99999907_m1, and ribosomal protein L13a Hs04194366_g1.

#### ALP activity

The ALP activity was determined using a colorimetric end-point assay measuring the enzymatic conversion of P-nitrophenyl phosphate (Sigma) to the yellowish product P-nitrophenol in the presence of ALP. To each 30 μL solution from the cellular–scaffold construct after sonication, 70 μL 2-amino-2-methyl-propanol buffer and 100 mL P-nitrophenyl phosphate (4 mg/mL) were added. Samples were incubated for 15 min at 37°C, and the reaction was stopped by the addition of 100 μL 2 M NaOH. Absorbance of P-nitrophenol was measured by a microplate reader (Victor3 1420 Multilabel Counter; PerkinElmer Life Sciences) at wavelengths of 405 and 600 nm. Standards were prepared from P-nitrophenol (concentration range: 0–0.2 mM). Technical duplicates were used for each biological sample. The ALP activity was expressed as nmol Nitrophenol/mL × min/μg DNA.

#### Calcium contents assay

Calcium contents were quantified by the colorimetric end-point assay based on the complexation of one Ca^2+^ ion with two Arsenazo III molecules to a blue-purple product (Diagnostic Chemicals Limited). Briefly, the calcium deposition in the cellular–scaffold construct was dissolved in 1 M acetic acid by placing in a shaker overnight. The samples were diluted (1:10 on days 2, 7, 14, and 1:20 on day 21) with double-distilled H_2_O, and aliquots of 20 μL were transferred to a 96-well plate. Arsenazo III solution (280 μL/well) was added and incubated for 10 min at room temperature. A standard dilution series of calcium ranging from 0 to 50 μg/mL was prepared, and Ca^2+^ concentration was quantified spectrophotometrically at 650 nm. Calcium content was expressed as micrograms of Ca^2+^ per scaffold.

#### Alizarin Red staining

Alizarin Red staining was used to visualize mineralization. Scaffolds from each group were fixed in 70% ethanol, embedded in Technovit 7100, and vertically cut in sections of 25 μm. Sections were then stained with 0.2% Alizarin Red R (Sigma A5533), and pictures were taken from the central part of the scaffold using a BX50 microscope with a Camedia C-5060 camera.

### Statistical analysis

Results are presented as mean ± SD for *n* = 3 biological replicates. Statistics were assessed using Stata 10.0 (StataCorp). Data from the different experiments were checked by QQ-plots. Interactions were examined using two-way analysis of variance (ANOVA; time × scaffold type). When significant main effects or an interaction between main effects were found, specific comparisons were made with Student's *t*-tests (equal variance) or the Wilcoxon rank-sum test (nonequal variance). One-way ANOVA was used to compare the difference of DNA amount within the same type of scaffold at different time points. Differences between means were considered statistically significant when *p*-values <0.05.

## Results

### Scaffold morphology

**FIG. 1.**
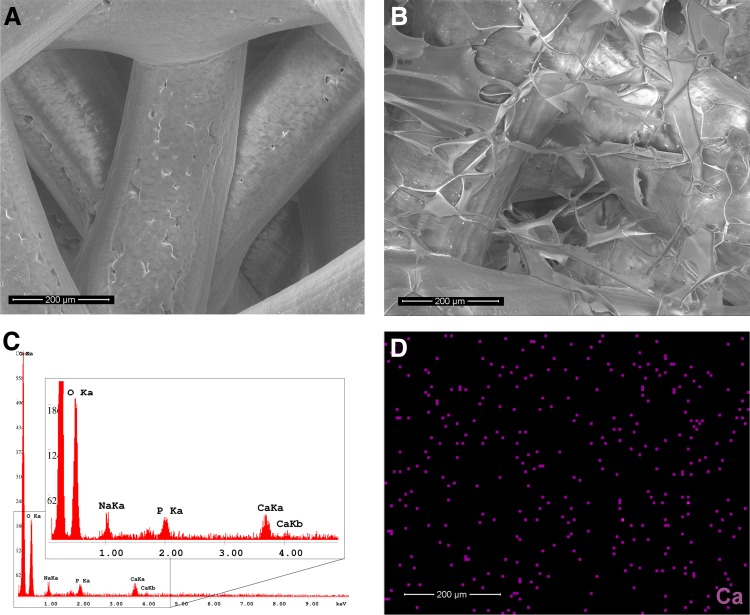
SEM micrographs and EDA of the scaffolds without cell culture. **(A)** SEM picture of PCL scaffold with highly interconnected pores. **(B)** SEM picture of HA/TCP-PCL scaffold. **(C)** EDA of HA/TCP-PCL scaffold. **(D)** The distribution of Ca element within HA/TCP-PCL scaffold. Bar = 200 μm. EDA, energy dispersive X-ray spectrometer analysis; HA/TCP, hyaluronic acid/β-tricalcium phosphate; PCL, polycaprolactone; SEM, scanning electron microscope.

Porous PCL scaffolds were fabricated by FDM (Fig. 1A). The deposited fibers were ellipsoidal in their cross section having semiaxes of 170 and 120 μm, respectively. The thickness of each individual layer was consequently also 120 μm. The center–center fiber distance in each deposited layer was 1.0 mm, and the fiber orientation of each consecutive layer was angled 105° and shifted 0.17 mm. SEM analysis showed that the scaffolds were completely coated with a HA/TCP layer on the PCL fibers as well as in the pores between the fibers (Fig. 1B). EDX analysis and EDX mapping of the element components indicated that the TCP was dispersed uniformly on the scaffold-coating layer (Fig. 1C, D).

### Cell distribution and morphology within the scaffolds

**FIG. 2.**
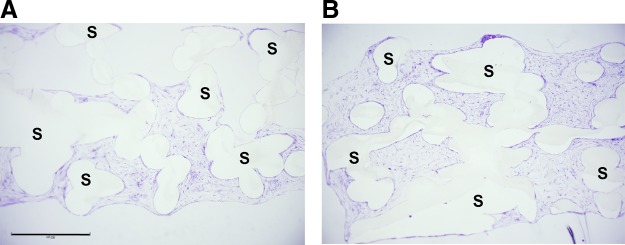
Toluidine blue stained cross sections of scaffolds in the Z-direction from top to bottom after 21 days of culture on **(A)** PCL scaffold and **(B)** HA/TCP-PCL scaffold. Cells only covered on the bottom part of the PCL scaffold, while cells were distributed uniformly on the HA/TCP-PCL scaffold. Scale bar = 900 μm. S, scaffold.

Toluidine blue stained cross sections of the scaffolds in the Z-direction from top to bottom showed that cell distribution in the PCL scaffold was not uniform after 21 days of culture. Only the bottom part of the scaffold was covered with cells (Fig. 2A). In the HA/TCP-PCL scaffold, cells were uniformly distributed in the entire scaffold after 21 days of cell culture (Fig. 2B).

**FIG. 3.**
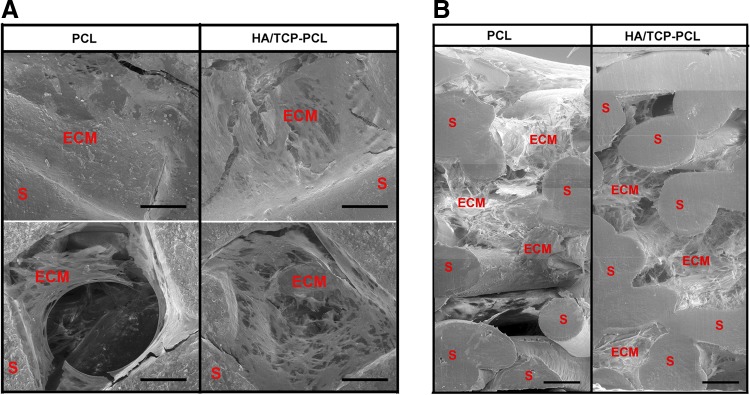
SEM images of scaffolds after 21 days of culture. **(A)** Both surfaces of cell seeding side and bottom side of the PCL and HA/TCP-PCL scaffolds with cell cultures. **(B)** Stitched cross sections of middle parts of PCL and HA/TCP-PCL scaffolds from the top to the bottom. Bar = 200 μm. ECM, extracellular matrix.

SEM images of the scaffolds after 21 days of culture showed that both the cell seeding side and the bottom side of the PCL and HA/TCP-PCL scaffolds were covered with layers of cells and matrix, whereas the PCL scaffold was fully covered on one side of the scaffold while the other side was still uncovered (Fig. 3A). The cross sections gave an overview of the cell distribution and matrix morphology inside the scaffolds (Fig. 3B). For the PCL scaffolds, the cells and matrix mainly covered part of the scaffold and the matrix was either compact as a cellular sheet or with a fluffy appearance between the fibers of the scaffold. For the HA/TCP-PCL scaffold, the cell matrix was spread out uniformly and attached to the fibers of the scaffold.

### DNA quantification

**FIG. 4.**
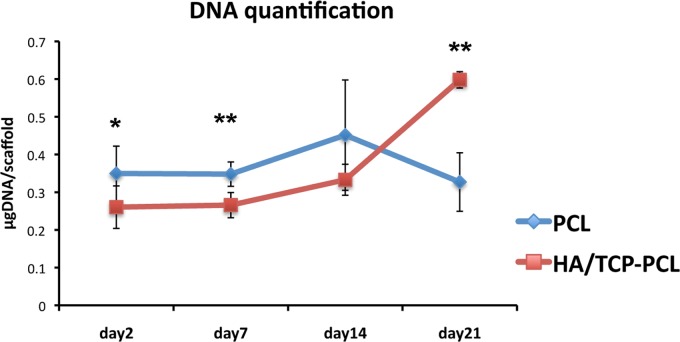
Quantification of dsDNA in cellular scaffolds by PicoGreen assay on days 2, 7, 14, and 21. The amount of DNA is expressed as mean ± SD (*n* = 3). Significant difference between PCL and HA/TCP-PCL scaffolds within the same culture condition and time point (*p < 0.05; **p < 0.01).

The DNA quantification, assumed proportional to the cell number, revealed the cell proliferation in the scaffolds over time (Fig. 4). There is no significant difference in the DNA amount within different time points for the PCL scaffold by the one-way ANOVA test. While cell number significantly (*p* < 0.001) increased on day 21 compared to earlier time points for the HA/TCP-PCL scaffold. Even though there was a higher initial cell number in the PCL scaffold compared to the HA/TCP-PCL scaffold on day 2 (*p* = 0.038), the cell number was significantly higher in the HA/TCP-PCL scaffold than the PCL scaffold after 21 days of culture (*p* = 0.004).

### Osteogenic differentiation of hMSCs within the scaffolds

**FIG. 5.**
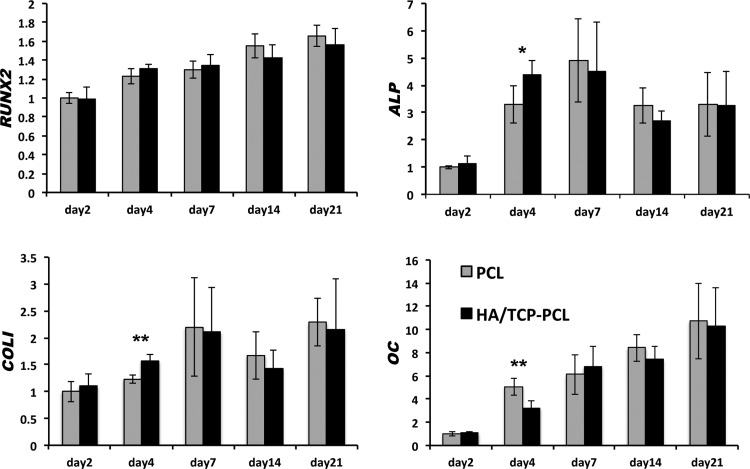
Gene expression of *RUNX2*, *ALP*, *COLI*, and *OC* after osteogenic culture of human mesenchymal stem cells on PCL and HA/TCP-PCL scaffolds up to 21 days. Vertical axes represent the BestKeeper relative gene expression relative to day 2 of the PCL scaffold for *RUNX2*, *ALP*, *COLI*, and *OC*. Horizontal axes represent the time points. Data are expressed as mean ± SD (*n* = 6). Significant difference between PCL and HA/TCP-PCL scaffolds at each time point (*p < 0.05; **p < 0.01).

Gene expression analysis by RT-qPCR showed that HA/TCP-PCL coating significantly increased *ALP and COLI* expression on day 4. *OC* expression was decreased on day 4 by HA/TCP coating. *RUNX2* expression was not affected by the HA/TCP coating. There is no significant difference of *RUNX2, ALP, COLI*, and *OC* expression after day 7 (Fig. 5).

**FIG. 6.**
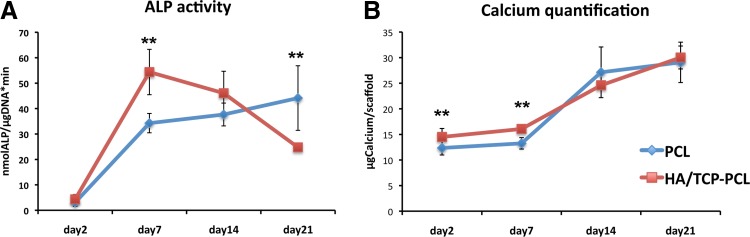
Osteogenic differentiation of scaffold/cell constructs. **(A)** Activity of the ALP enzyme on days 2, 7, 14, and 21. The activity is expressed as mean ± SD (*n* = 3). Activity is indicated in nanomole p-nitrophenol/microgram DNA per minute (nmol/mL * μgDNA*min). **(B)** Calcium contents per scaffold on days 2, 7, 14, and 21. The amount of calcium is expressed as mean ± SD (*n* = 3). Significant difference between PCL and HA/TCP-PCL scaffolds within the same culture condition and time point (**p < 0.01). ALP, alkaline phosphatase.

The ALP activity of the cells cultured in the two types of scaffolds had a different tendency (Fig. 6A). For the PCL scaffold, the ALP activity increased from day 2 to 21, while for the HA/TCP-PCL scaffolds, the ALP activity peaked on day 7 and then decreased. The ALP activity of the HA/TCP-PCL scaffolds was 1.6 times higher compared with PCL scaffolds on day 7 (*p* = 0.001), while the ALP activity of the PCL scaffold was 1.8 times higher compared with HA/TCP-PCL scaffolds on day 21 (*p* = 0.004).

To assess the extent of matrix mineralization in the scaffolds, the calcium content was determined (Fig. 6B). There was an initial higher amount of calcium in the HA/TCP-PCL scaffolds compared to the PCL scaffolds on day 2. On day 7, the HA/TCP-PCL scaffolds contained a significantly higher amount of calcium compared to the PCL scaffolds. The calcium amount increased significantly from day 14 to 21 for both types of scaffolds, but with no significant difference between the two groups.

**FIG. 7.**
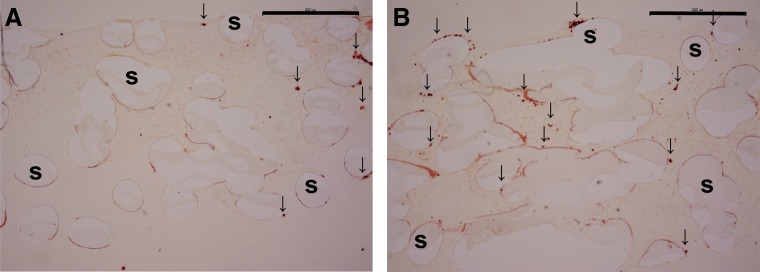
Alizarin Red stained cross sections of scaffolds with 21 days cell culture on **(A)** PCL scaffold and **(B)** HA/TCP-PCL scaffold. Scale bar is 900 μm. Representative positive staining of calcium was pointed out with an arrow.

Alizarin Red staining of the scaffolds revealed the calcium deposition in both the PCL and HA/TCP-PCL scaffolds after 21 days of culture (Fig. 7). Small round Alizarin Red-positive nodules formed more uniformly in the HA/TCP-PCL scaffold compared to the PCL scaffold.

## Discussion

In this study, we showed that HA/TCP composite coating of PCL scaffold improved cellular proliferation and osteogenic differentiation of hMSCs and uniform cellular matrix deposition within the scaffold.

In the present study, it was shown that cells were more uniformly distributed in the HA/TCP-PCL scaffolds compared to the PCL scaffolds after 21 days of culture. This result was comparable with our earlier study with human dental pulp stem cells,^17^ which we showed that cells clustered into small groups toward the bottom of the PCL scaffold on day 1. After day 1, cells proliferated from these clustered areas to form lager clusters throughout the scaffold on days 7, 14, and 21. Cells were more evenly dispersed in the HA/TCP-PCL scaffold from day 1, and then cell density was increased and evenly distributed throughout the entire scaffold on day 21. This was mainly a consequence of the geometry of the scaffolds where the pore size of the PCL scaffold was much bigger than the size of an MSC. During seeding of the cells, the interior of the scaffolds becomes wetted by the seeding suspension and due to capillary forces, the cell suspension volume is drawn into the scaffold. During the period between adding the cell suspension and adding the remainder of the cell culture media, cells will gradually settle on the interior fiber surfaces in the scaffolds. However, due to gravity and the open pore geometry, a substantial amount of cells will enter the scaffold to a considerable depth before either attaching in the lower part of the scaffold or completely escaping the scaffold. Unattached cells will subsequently die and be washed out during medium change.

In this study, HA was deliberately embedded in the scaffold without subsequent crosslinking. This causes the porous HA/TCP foam structure to intermittently act as a hydrophilic sponge before dissolving into a viscous solution following seeding with cells. These effects help to distribute cells evenly into the scaffold and the increased viscous resistance effectively negates the sedimentation effect present in the PCL scaffold and together result in an even cell seeding. Omitting crosslinking, the HA/TCP matrix avoids the risk of creating a permanent porous matrix with too small micropores that would impede cell movement and also avoids issues with cross-linker cytotoxicity.^20^

In addition to the physical effects of a HA sponge, HA improves cellular infiltration and stimulates MSC migration.^21^ HA is a very large glycosaminoglycan and has unique hygroscopic, rheological, and viscoelastic properties,^22^ which might prevent cells from firmly attaching onto scaffold fibers and lead to increased cell migration and cellular matrix formation.^23,24^ Cells respond to HA through cell surface binding proteins such as CD44 and RHAMM leading to enhanced cellular mobility, attachment strength, and survival.^25,26^

In the current study, it was shown that there is no significant difference in cell number for the PCL scaffold within 21 days of culture time, while cell number significantly increased on day 21 for the HA/TCP-PCL scaffold. This result was different with our earlier study on the PCL scaffold, where we observed that cell number was increasing during culture time.^17^ This might be because cells were in osteoinduced culture from day 1 in the present study, while in the other study, cells were in noninduction culture for 1 week before osteoinduced culture. Another study from Leong et al. also showed that DNA amount of adipose tissue-derived stem cells increased significantly for noninduced culture between days 14 and 28, while there was no similar increase for the osteoinduced culture.^27^

The cell number was significantly increased in the HA/TCP-PCL scaffold on day 21. This advantage could come from the coating components HA and TCP. It has been shown that incorporating β-TCP into scaffolds could significantly increase the proliferation of stem cells,^28–30^ and HA was shown to enhance the proliferation of mouse and porcine MSCs.^31–33^ The difference of DNA amount between the PCL and HA/TCP-PCL scaffold on days 2 and 7 might be owing to different initial cell attachment. We have previously shown that HA/TCP-PCL scaffold has lower cell seeding efficiency than the PCL scaffold.^17^ This result was conflicting with our hypothesis that increasing the surface and roughness of a scaffold would improve initial cell attachment, which has also been shown in other studies.^34–36^

Gene expression and calcium quantification results showed that HA/TCP scaffolds stimulated osteogenic differentiation of hMSCs but not the mineralization. Studies have shown that TCP increased the ALP activity and osteocalcin content of stem cells *in vitro* and promoted contact osteogenesis *in vivo*.^28–30^ It was also reported that HA upregulated *ALP* and *OC* expression and had an effect on the increasing calcium deposit of porcine MSCs at day 21.^33^ Another study also reported that HA has a molecular weight-specific and dose-specific mode of action that may enhance the osteogenic and osteoinductive properties.^32^

Our gene expression results showed that HA/TCP coating significantly increased the mRNA expression of *ALP* and *COLI* on day 4. These data correlated with the data obtained from the biochemical assays for ALP activity. For the HA/TCP-PCL scaffolds, the *ALP* peak on day 4 correlated with the ALP peak on day 7, while for the PCL scaffold both the gene and protein were increasing during culture time. However, HA/TCP coating did not increase the mineralization of hMSCs in the present study. Gene expression of *OC* was decreased by HA/TCP coating at day 4. There is no significant difference of *RUNX2, ALP, COLI*, and *OC* expression between the PCL and HA/TCP-PCL scaffolds after day 7.

Calcium quantification data showed that HA/TCP-PCL contained higher calcium amount only the first week, but there was no significant difference between the HA/TCP-PCL and PCL groups on days 14 and 21. The advantage of HA/TCP coating for stimulating mineralization of hMSCs in this study was not the same compared to our earlier studies with other stem cell sources. For human dental pulp stem cells, HA/TCP coating significantly increased the Ca^2+^ content on both days 14 and 21^17^; for mesenchymal stem cells derived from human induced-pluripotent stem cells (iPS-MSCs), HA/TCP coating significantly increased the Ca^2+^ content on day 21.^37^ This suggested that the osteogenic differentiation capacity of human dental pulp stem cells and human iPS-MSCs might be higher than the hMSCs. Individual variation from different donors, however, must also be taken into consideration. There are many other studies that reported significant osteogenic differentiation of hMSCs when cultured on materials containing calcium phosphates.^38,39^ Since, in our study, we used only 0.4 mg/mL of TCP for coating the PCL scaffold, this amount could be too little to have a significant osteogenic differentiation of hMSCs. To improve the coating system, we could increase the amount of TCP.

However, from the qualitative histology data of mineralization, it seemed that there were more uniform mineralization deposits within the HA/TCP-PCL scaffold, especially in the center of the scaffold. We speculated whether the calcium quantification assay was not optimized; maybe still some calcium deposits were not completely dissolved in the acid solution. Another explanation for the similarity between the two groups on days 14 and 21 could be that the transport of Ca^2+^ and PO_4_^3−^ from the culture medium to the interior of the scaffold was diffusion limited. At the later time points when cells had proliferated and reached confluence, ion transport becomes more restricted and mineralization rates will be primarily determined by this and less by enzyme activity. This reflects the limitation of static cell culturing and it will be interesting in the future to test the scaffolds in dynamic cell culture setups, such as spinner flask or perfusion systems.^40–45^

In this study, the intraexperiment variation was kept low using characterized commercially available hMSCs from a single donor. While the osteogenic effects of the HA/TCP modifications could be different on MSCs from different donors,^46^ it can be assumed that the advantageous cell distributive effects arising from the coating's sponge-like properties will remain the same. We have previously shown that the scaffold promotes migration and osteogenic differentiation of human dental pulp stem cells.^17^

## Conclusions

This *in vitro* study investigated some important yet often overlooked issues in basic tissue engineering: How can seeding efficiency and homogeneity of a limited stem cell resource be improved at the bench? Furthermore, addressing the area of bone tissue engineering, we wanted to demonstrate a means of improving *in vitro* osteoconductivity of polymeric scaffold.

We used a natural polymer in the form of the extracellular matrix component HA and a ceramic material of TCP to functionalize a PCL scaffold made by FDM. Despite the simple composition and lack of crosslinking, our results showed that this coating stimulated the proliferation and osteogenic differentiation of hMSCs. Cellular matrix and calcium deposition were uniformly distributed in the coated scaffold. Considering that all the scaffold's constituents are safe and FDA approved, these results demonstrated that the HA/TCP-PCL scaffolds hold great promise to accommodate human bone marrow-derived MSCs for bone reconstruction purposes, which warrants future *in vivo* studies.

## Abbreviations Used

ANOVA: analysis of variance
BTE: bone tissue engineering
cDNA: complementary DNA
DMEM: Dulbecco's Modified Eagle's Medium
EDX: energy dispersive X-ray spectrometer
FBS: fetal bovine serum
FDM: fused deposition modeling
HA/TCP: hyaluronic acid/β-tricalcium phosphate
hMSC: human mesenchymal stem cell
PBS: phosphate-buffered saline
PCL: polycaprolactone
RT-qPCR: reverse transcription quantitative polymerase chain reaction
SEM: scanning electron microscope
